# Neutralizing antibody levels as a key factor in determining the immunogenic efficacy of the novel PEDV alpha coronavirus vaccine

**DOI:** 10.1080/01652176.2025.2509506

**Published:** 2025-05-28

**Authors:** Guangli Hu, Xin Luo, Jiamin Liao, Chuangchao Zou, Yihui Huang, Rui Geng, Zhiqing Zhao, Hanqin Shen, Yongchang Cao, Ouyang Peng, Hao Zhang

**Affiliations:** a State Key Laboratory of Biocontrol, School of Life Sciences, Sun Yat-sen University, Guangzhou, China; b Guangdong Provincial Enterprise Key Laboratory of Healthy Animal Husbandry and Environment Control, Wen’s Foodstuff Group Co. Ltd, Yunfu, China

**Keywords:** Porcine epidemic diarrhea virus, coronavirus, neutralizing antibody, vaccines, mutant strain, S protein, equivalent epitope, broad protection

## Abstract

Porcine epidemic diarrhea virus (PEDV) causes significant global agricultural losses. Despite commercial inactivated and live attenuated vaccines, persistent outbreaks underscore the need for more effective solutions. Here, we isolated a novel Chinese PEDV variant, PEDV ShXXY2-2023, with amino acid substitutions in key neutralizing epitopes (N-terminal domain, receptor-binding domain, and CO-26K equivalent epitope) compared to vaccine strains. An inactivated ShXXY2-2023 vaccine induced higher neutralizing antibodies and superior cross-protection versus commercial vaccines. Vaccinated sows conferred enhanced protection to offspring, improving piglet survival post-challenge. Maternal serum neutralizing antibody titers correlated strongly with piglet survival; titers of 1:377–1:774 at one week prepartum yielded >80% protective efficacy. These findings emphasize neutralizing antibodies’ critical role in PEDV prevention and position ShXXY2-2023 as a promising vaccine candidate, with broader implications for coronavirus vaccine development.

## Introduction

Coronaviruses (CoVs) belong to the Coronaviridae family and are enveloped viruses with a single-stranded positive-sense RNA genome of approximately 26–32 kilobases, which is the largest known RNA viral genome. This group of viruses has caused several severe epidemics in humans and animals, including the Severe Acute Respiratory Syndrome Coronavirus 2 (SARS-CoV-2), which first emerged in Hubei Province, China, in late 2019. Since then, SARS-CoV-2 has infected more than 115 million people worldwide and has resulted in over 2.5 million deaths (Dong et al. 2020; Zhou et al. 2020; Zhu et al. 2020). Since the early 1970s, various pathological conditions in domestic animals have been attributed to CoV infections (Durham et al. 1979). A alphacoronavirus, porcine epidemic diarrhea virus (PEDV), first emerged in European swine herds. PEDV is a highly contagious intestinal disease characterized by vomiting, diarrhea, and dehydration in pigs of all ages (Pensaert and de Bouck 1978; Madson et al. 2014). Infections occur across all age groups, with piglets being the most severely affected, exhibiting morbidity and mortality rates of up to 100% (Madson et al. 2014). The virus was successfully isolated in the United Kingdom in 1978 (Wood 1977). Severe outbreaks with highly virulent PEDV strains have since spread to many regions, including China, Japan, Korea, Thailand, Canada, Mexico, and the United States, resulting in annual economic losses of hundreds of millions of dollars and causing significant damage to the pig industry (Sun R-Q et al., 2012; Jung and Saif 2015; Ojkic et al. 2015; Yamamoto et al. 2015).

The primary method for preventing and controlling coronavirus outbreaks is vaccination (Langel et al. 2016; Si et al. 2020; Liu Q and Wang 2021; Xia et al. 2021; Zhang Y et al. 2021). The spike (S) glycoprotein on the surface of the virion plays a pivotal role in mediating viral attachment and entry into host cells. It is a trimeric transmembrane protein composed of two functional subunits: S1 and S2. The S1 subunit contains the N-terminal domain (NTD) and receptor-binding domain (RBD), both of which are responsible for binding to host cell receptors. The S2 subunit facilitates membrane fusion, allowing viral entry into the host cell (Li F 2016; Li W et al. 2016). S proteins contain multiple neutralizing epitopes and serve as major targets for neutralizing antibodies. They are also key immunogens and ideal antigenic targets for vaccine development (Chang SH et al. 2002; Oh et al. 2014; Liu L et al. 2020; Vanhove et al. 2021). However, the S protein is a mutational hotspot, where adaptive mutations can enhance transmissibility, infectivity, and host immune evasion (Dorp et al. 2020; Li Q et al. 2020; Zhang H et al. 2023). Serum-neutralizing antibodies produced by current vaccines are significantly less effective against the mutant strain, although this has yet to be confirmed in clinical trials (Dejnirattisai et al. 2021; Heath et al. 2021; Parry et al. 2021; Sapkal G et al. 2021; Sapkal GN et al. 2021; Wu et al. 2021; Schubert et al. 2022; Yu et al. 2022). The key question is whether these mutations have a tangible impact on the pathogenicity of the coronavirus. This understanding is crucial for elucidating viral infection mechanisms and shaping strategies for drug and vaccine development in preparation for the next stage of the pandemic (Yao et al. 2020).

Despite the widespread use of commercial inactivated and live attenuated vaccines for the prevention and control of PEDV, several critical limitations persist. For instance, most current vaccines are derived from early epidemic strains and fail to provide sufficient protection against newly emerging, highly variable strains (Lee C 2015). Moreover, the frequent mutations occurring in the spike (S) protein of PEDV exacerbate the growing antigenic mismatch between vaccine strains and circulating field strains, thereby further diminishing the cross-protective efficacy of existing vaccines (Sun R-Q et al. 2012). In this study, we isolated a strain called PEDV ShXXY2-2023 from a swine farm with PEDV outbreaks. Compared to the vaccine strain and PEDV GDS01 strain, the PEDV ShXXY2-2023 strain exhibits amino acid substitutions in critical regions of the S1 subunit, including the N-terminal domain (NTD) and receptor-binding domain (RBD), which are thought to enhance immune evasion and may reduce the effectiveness of existing vaccines. We formulated this strain into an inactivated vaccine and assessed its immunogenicity. The PEDV ShXXY2-2023 vaccine induced significantly higher levels of neutralizing antibodies compared to current commercial vaccines, resulting in a markedly improved survival rate in challenged piglets (88% vs. 52–60%). A positive correlation was observed between the serum neutralizing antibody levels in sows one week before farrowing and the survival rate of piglets during challenge tests, highlighting the importance of neutralizing antibodies in protecting piglets. When pregnant sows exhibited serum neutralizing antibody titers ranging from 1:377 to 1:744 or higher one week prior to farrowing, the survival rate of their offspring following PEDV infection reached over 80%, indicating a strong protective effect conferred by maternal immunity. This result is significant for PEDV control and evaluating vaccine efficacy. It also establishes an effective evaluation system for preventing PEDV infections, where high levels of neutralizing antibodies are identified as a critical factor for protection against PEDV infections.

## Methods

### Clinical sample collection and cells

A total of 21 piglet intestinal samples were collected from a PEDV-affected pig farm in Shanxi Province, China, in January 2023. The virus isolated from these samples was identified as PEDV using N gene-based reverse transcription qPCR (RT-qPCR). The intestinal content samples were homogenized in serum-free Dulbecco’s Modified Eagle’s Medium (DMEM; Invitrogen, USA) with 1% penicillin-streptomycin (10,000 units/mL penicillin and 10,000 μg/mL streptomycin; Gibco^™^, USA) and 0.3% trypsin phosphate broth (TPB; Sigma, Germany), then centrifuged at 1000 rpm for 10 min at 4 °C. The supernatant was filtered through a 0.22-μm pore size filter (Merck Millipore, Germany) to remove bacteria and was subsequently frozen at −80 °C until used as an inoculum for virus isolation. Vero cells (ATCC CCL-81) were cultured in alpha-minimum essential medium (α-MEM; Invitrogen, Carlsbad, CA) supplemented with 5% fetal bovine serum (FBS; Invitrogen) and 100 × antibiotic-antimycotic solution (Invitrogen), and maintained at 37 °C in a humidified 5% CO_2_ incubator.

### Isolation and serial passaging of the virus

A 100% confluent Vero cell monolayer was washed with sterile phosphate-buffered saline (PBS; pH 7.2, Gibco^™^, USA) three times to thoroughly remove fetal bovine serum (FBS). In addition, the supernatants of the intestinal content samples prepared as described above were diluted five-fold in virus growth medium [DMEM supplemented with antibiotics (100 units/mL penicillin and 100 mg/mL streptomycin, Gibco^™^) and 10 μg/mL trypsin (Gibco^™^, USA)], vortexed briefly and subsequently used as an inoculum. A total of 1 mL of the prepared inoculum was added to a T-25 flask. After incubation at 37 °C for 2 h, 2 mL of virus growth medium was added without removing the inoculum. The inoculated cells were maintained at 37 °C in a humidified 5% CO2 incubator and monitored daily for cytopathic effects (CPEs). When a CPE was observed in more than 90% of Vero cells, the flask was subjected to three rounds of freezing and thawing. The cells and supernatants were mixed by pipetting, aliquoted and stored at −80 °C. These harvested cells were used as the seed stock for the next passage. For serial passaging, the culture scale was gradually increased until T-75 flasks were used for propagation and serial passaging of the PEDV strains. The isolated strain was designated as PEDV ShXXY2-2023 (GenBank accession no. PQ316092). PEDV strain GDS01 (GenBank accession no. KM089829) was isolated and maintained in our laboratory as previously described.

### Virus titration

Vero cells were seeded into 96-well plates, and after reaching confluence, the monolayers were washed three times with PBS (Gibco^™^, USA). In addition, 100 μL of 10-fold serially diluted virus suspensions containing 10 μg/mL trypsin were inoculated into eight replicates per dilution. After absorption for 2 h, another 100 μL of virus growth medium was added to each well. The viral CPE was monitored for three to five days, and the viral titers were calculated according to the Reed and Muench method and expressed as TCID_50_/mL.

### Electron microscopy

To image virion particles in Vero cell culture medium, Vero cells infected with PEDV were harvested when a CPE was observed in more than 90% of the cells. The cell culture mixture was frozen and thawed three times and then centrifuged at 10,000 rpm at 4 °C for 1 h. The supernatant was filtered through a 0.22-mm filter to remove the cell debris and then mixed with polyethylene glycol 8000 (PEG-8000; Solarbio, China) to a final concentration of 10% overnight. The mixture was then ultracentrifuged at 12,000 rpm at 4 °C for 2 h to pellet the PEDV particles. The viral particle pellets were resuspended in Tris-buffered saline (TBS), negatively stained with 2% phosphotungstic acid and examined with a transmission electron microscope (JEOL, JEM-1200EX, Japan).

### Immunofluorescence assay (IFA)

Vero cells in six-well cell culture plates were mock infected or infected with PEDV at a multiplicity of infection (MOI) of 0.1. At 0, 12, 24 and 36 h post-infection, the cells were fixed with 4% paraformaldehyde at 4 °C for 30 min and then permeabilized with 0.25% Triton X-100 (Solarbio, China) for 10 min at room temperature (RT). After the plate was washed three times with PBS (Gibco^™^, USA), it was blocked with 5% bovine serum albumin (BSA; Solarbio, China) at RT for 1 h. Mouse anti-PEDV S protein 2C10 (preserved in this laboratory) and Alexa Fluor^®^ 488-conjugated goat anti-mouse IgG (Abcam, UK) were used as the primary and secondary antibodies, respectively. The cell nuclei were stained with 4′,6-diamidino-2-phenylindole (DAPI; Vectorlabs, USA) for 5 min at RT. After the cells were washed with PBS, the stained cells were observed with a fluorescence microscope (Olympus, Japan).

### RNA extraction, library construction, and sequencing

RNA was extracted from clarified infected cell lysate samples or purified virus using TRIzol and prepared for next-generation sequencing. Briefly, reverse transcription was performed using random hexamers. Subsequent DNase treatment and cleanup were followed by second-strand synthesis, after which library preparation was conducted using Nextera XT reagents (Illumina) and sequencing was performed on the NovaSeq 6000 platform (Illumina). Although originally described as a consensus-level sequencing methodology, the depth of coverage was sufficient to perform deep sequencing analysis as well. Bioinformatics analysis of the data was performed using the previously described pipeline.

### Genome sequencing, assembly, and annotation

Raw reads were filtered and trimmed by fastp (https://github.com/OpenGene/fastp) to remove sequencing adapters and low-quality reads, including those reads scored < Q20. Ribosomal RNAs and host reads subtraction by read-mapping were performed with BBMAP program. De novo genome assembly was performed using SPAdes v3.13.0 (Nurk et al. 2013). These extracted assembled scaffolds limited the minimum contig length to 100 bases, with the best BLAST hits to NCBI nt database.

### Phylogenetic analysis of the spike (S) gene

The sequence fragments were assembled and analyzed with DNAStar 7.0 and BioEdit software, respectively. All of the complete sequences of the S gene and reference sequences obtained from GenBank were used for sequence alignments and phylogenetic analyses. Phylogenetic trees were constructed *via* the neighbor–joining method and using MEGA version 6, with bootstrap values calculated for each node from 1000 replicates. All tree figures were produced using MEGA 4.0 software.

### Neutralization assays

The collected serum samples were heat inactivated for 30 min at 56 °C and serially diluted in twofold increments. The diluted samples were then mixed with an equal amount of PEDV (200 TCID_50_) and incubated at 37 °C for 1 h. Subsequently, 0.1 mL of each mixture was transferred to a Vero (monkey kidney) cell monolayer, which was cultured in a 96-well tissue culture plate and washed once with DMEM before PEDV application. After adsorption for 1.5 h at 37 °C, the inoculum was discarded, and the cells were washed twice with PBS. Next, maintenance medium containing trypsin (10 µg/mL) was added to each well, and the plate was incubated for 48 h at 37 °C. The cells were examined daily for CPEs. The neutralizing antibody titers are expressed as the highest serum dilution that protected more than 50% of the cells from CPE.

### B-cell epitope prediction

Immunoinformatics tools were exploited to predict B-cell epitopes and the properties of the amino acid residues. First, linear B-cell epitope prediction was performed *via* BepiPred-2.0 (http://www.cbs.dtu.dk/services/BepiPred/), which predicts linear B-cell epitopes from a protein sequence *via* a random forest algorithm trained on epitope and nonepitope amino acids determined from crystal structures, followed by sequential prediction smoothing. In our study, the residues with a threshold score of epitope probability above 0.5 were considered parts of a B-cell epitope. At 0.5, it shows 65.93% accuracy. An increase in the threshold results in better specificity. Putative B-cell epitopes were selected on the basis of the region with at least six consecutive residues predicted to have an epitope probability above 0.5. Notably, BepiPred-2.0 also predicts and provides the accessibility and coil probability of each amino acid residue. In addition, other properties of the proteins were also characterized *via* multiple immunoinformatics tools provided by the IEDB (http://tools.iedb.org/bcell/), except IUPred. IUPred (https://iupred.elte. hu) was used to predict intrinsically unstructured proteins, which infers the coil probability.

### Homology three-dimensional modeling

The 3D structure of the S protein was analyzed *via* the SWISS MODEL server *via* homology modeling. The server automatically performs a BLASTp search to identify templates for each protein sequence. From the query results, the template protein 6u7k was selected for homology modeling. This is an atomic-resolution structural model of the PEDV spike protein with 80.12% sequence identity, which is a reliable score for initiating modeling. The resulting docked complexes were visualized *via* the PyMOL molecular graphics system.

### ELISA for the detection of PEDV-specific antibodies

To measure the quantity of PEDV-specific antibodies *in vivo*, the concentrations of PEDV-specific IgA antibodies in the serum or in the colostrum were detected *via* commercial enzyme-linked immunosorbent assay (ELISA) kits (IDEXX, America) in accordance with the manufacturer’s instructions. The PEDV-specific IgG antibodies in serum or colostrum were detected *via* commercial ELISA kits (Biao Yun Bio, China) in accordance with the manufacturer’s instructions. For IgA and IgG detection, a sample-to-positive (S/P) ratio ≥ 0.5 was considered to indicate positivity, and a S/P ratio < 0.5 was considered to indicate negativity. The S/P ratio was calculated as follows: S/P = (sample Mean – NC)/(PC – NC).

### Viral real-time quantitative PCR (RT–qPCR)

Total RNA was extracted *via* an RNeasy Mini Kit (Qiagen), and reverse transcription was conducted *via* a Super Script III First-Strand Synthesis Kit (Invitrogen). TaqMan qPCR was performed for PEDV in a 96-well optical plate (Applied Biosystems) at 95 °C for 10 min, followed by 40 cycles of 95 °C for 30 s, 60 °C for 30 s, and 72 °C for 30 s. The sequences of the primers and probes for TGEV and PEDV31 were as follows: PEDV: forward: 5′-GAATTCCCAAGGGCGAAAAT-3′, reverse: 5′-TTTTCGACAAATTCCGCATCT-3′, and probe: 5′-CGTAGCAGCTTGCTTCGGACCCA TAC-3′. The purified PEDV genomic RNAs were used to generate a standard curve in RT–qPCR assays.

### Commercial inactivated PEDV vaccines

To evaluate the immunogenicity and protective efficacy of currently available commercial vaccines, three inactivated porcine epidemic diarrhea virus (PEDV) vaccines were purchased from licensed Chinese veterinary biological product manufacturers. For the purpose of this study, and to ensure anonymity of commercial sources, the vaccines were designated as Vac A, Vac B, and Vac C, respectively.

All three vaccines were based on the G2b genotype variant strains of PEDV, which are widely used in China for immunization against field-circulating epidemic strains. The vaccines were stored and handled according to the manufacturers’ instructions, and were administered intramuscularly to experimental animals at the dosage recommended by the corresponding product labels.

### Preparation of the experimental inactivated vaccines

The twelfth passage (P12) of cell culture-adapted PEDV ShXXY2-2023 was chemically inactivated using binary ethyleneimine (BEI) or formaldehyde. In brief, 10^7^ TCID_50_/mL viruses were obtained from Vero cells cultured in T-75 flasks (Corning, USA). After three rounds of freezing and thawing, the liquid supernatant containing the viruses was collected *via* centrifugation and then inactivated by the addition of 0.2 M BEI to achieve a final concentration of 2 mM and incubated at 30 °C for 24 h. After the reaction, the remaining BEI was neutralized by the addition of 20% sodium thiosulfate. The final concentration of formaldehyde inactivator used was 0.2%, and the samples were incubated at 37 °C for 20 h. Inactivated vaccines were prepared by mixing BEI or formaldehyde-inactivated PEDV ShXXY2-2023 with Al(OH)_3_ (Sigma, Germany) and 201 adjuvant (Sino-Gene, China), respectively, according to the instructions and then storage at 4 °C for later use. In the active immunization, 35 4-week-old PEDV virus-free piglets were randomly divided into seven groups (with five piglets in each group) and housed in separate rooms. The immunization dose and schedule are shown in Table 1. Blood serum samples were collected from each piglet before vaccination end on days 7, 14, 21, 28 and 35 for antibody testing, in order to select the optimal inactivation method and adjuvant for subsequent experiments.

**Table 1.** Combination of different adjuvants and inactivation methods for the preparation of inactivated vaccine of strain PEDV ShXXY2-2023.

Group	VIRUS titer(log10 TCID50/mL)	Adjuvant of vaccines	Inactivation	Injection	The dose of immunity	Numbers
A1	7.0	201	Formaldehyde	Intramuscular neck	2mL	5
A2	7.0	Al(OH)_3_	Formaldehyde	Intramuscular neck	2mL	5
B1	7.0	201	BEI	Intramuscular neck	2mL	5
B2	7.0	Al(OH)_3_	BEI	Intramuscular neck	2mL	5
C1	Negative control	PBS	/	Intramuscular neck	2mL	5
C2	Negative control	201	/	Intramuscular neck	2mL	5
C3	Negative control	Al(OH)_3_	/	Intramuscular neck	2mL	5

### Assessment of the immunogenicity of the vaccines in four-week-old piglets

25 4-Week-old PEDV-free piglets were randomly divided into five groups (five piglets per group) and housed in separate rooms. The PEDV ShXXY2-2023 inactivated vaccines, along with three types of commercial inactivated G2 strain vaccines named Vac A, Vac B, and Vac C (2 mL per pig), were injected into the neck muscles of the piglets, whereas the control group received phosphate-buffered saline (PBS). Blood serum samples were collected from each piglet before vaccination and at 7, 14, 21, 28, 35, 42, 49, 56, 63, 70, 77, 84, 91 and 98 days for antibody testing.

### Pathogenicity of PEDV ShXX2-2023 in piglets

All experimental animals were obtained from a conventional breeding farm with a documented history of good health. The herd had neither been vaccinated against PEDV nor experienced any outbreaks of PED, and was confirmed to be negative for all porcine enteric viruses through diagnostic testing. Ten 3-day-old piglets were randomly assigned to two groups of five and housed in separate rooms. The piglets were fed commercial fresh milk five times daily and had unrestricted access to distilled water throughout the experiment. Before challenge, rectal swabs confirmed that the piglets were negative for major porcine enteric viruses (PEDV, transmissible gastroenteritis virus, porcine delta coronavirus, swine acute diarrhea syndrome coronavirus, and porcine rotavirus) *via* RT-qPCR. The two groups were then orally inoculated with either 1 ml of 1 × 10^6^ TCID_50_ of PEDV ShXXY2-2023 or 1 ml of DMEM (mock infection). Following inoculation, the piglets were monitored daily for clinical symptoms such as depression, slow movement, diarrhea, vomiting, and anorexia. Rectal swabs were collected daily to monitor fecal viral RNA shedding using quantitative real-time RT-qPCR targeting the PEDV N gene. All surviving pigs were euthanized at the end of the experiment (five days post-inoculation). The clinical significance score (CSS) was used to assess the severity of diarrhea based on fecal consistency following viral challenge. The scoring criteria were as follows: 0, normal and no diarrhea; 1, soft stool; 2, mildly fluidic feces; 3, moderately mucous-to-watery diarrhea; and 4, severe watery and projectile diarrhea.

### Experimental design for active immunization of pregnant sows and passive immunization and challenge tests for piglets

In the experiment evaluating passive immunity in pregnant sows and active immunity as well as challenge protection in piglets, four groups of pregnant sows were tested. Sows in the first three groups were monitored prior to vaccination to ensure similar levels of IgG, IgA, and neutralizing antibodies for subsequent experimentation. In the first group, 15 pregnant sows were randomly assigned to three groups of five sows each and housed in separate rooms. Five sows were injected with 2 mL of a commercial inactivated vaccine of a G2 strain or PBS into the neck muscle, according to the schedule shown in Figure S4(A). Ten newborn piglets were randomly selected from each sow. Two groups underwent oral challenge with the PEDV ShXXY2-2023 or PEDV GDS01 strain at a dose of 1 × 10^6^ TCID_50_ per piglet between three and five days of lactation respectively. In the second group, 30 pregnant sows were randomly assigned to six groups of five sows each and housed in separate rooms, as shown in Figure 4(A). Five sows were injected with 2 mL of commercial inactivated vaccines of G2 strains (Vac A, Vac B, Vac C, PEDV ShXXY2-2023 inactivated vaccine) or PBS into the neck muscle. Ten newborn piglets were randomly selected from each sow. All groups underwent oral challenge with the PEDV ShXXY2-2023 at a dose of 1 × 10^6^ TCID_50_ per piglet between three and five days of lactation. In the third group, 15 pregnant sows were randomly assigned to three groups of five sows each and housed in separate rooms, according to the schedule shown in Figure 5(A). Five sows were injected with 2 mL of PEDV ShXXY2-2023 inactivated vaccine or PBS into the neck muscle. Ten newborn piglets were randomly selected from each sow. All groups underwent oral challenge with the PEDV GDS01 strain at a dose of 1 × 10^6^ TCID_50_ per piglet between three and five days of lactation. In the fourth group, 20 pregnant sows were randomly assigned to four groups of five sows each and housed in separate rooms, as shown in Figure 8(A). Grouped by neutralizing antibody titer, the high-level antibody group had titers of 1:1024, 1:724, 1:400, 1:512 and 1:637 against the PEDV ShXXY2-2023, while the low-level antibody group had titers of 1:256, 1:200, 1:181, 1:128, 1:181, and the PBS group had neutralizing antibodies around 1:4. Ten newborn piglets were randomly selected from each sow. All groups underwent oral challenge with the PEDV ShXXY2-2023 at a dose of 1 × 10^6^ TCID_50_ per piglet between three and five days of lactation. Serum samples were collected from all sows before and at 14 and 28 days post- immunization for antibody monitoring (IgG, IgA, and neutralizing antibodies). Colostrum samples were collected at parturition for antibody testing. Three piglets per sow were selected for serum testing of IgA, IgG, and neutralizing antibodies before and 14 days post- challenge. Fecal swab samples were collected daily post-challenge to measure PEDV RNA load and assess fecal consistency scores. The survival rate of challenged piglets was recorded for each group.

### Intestinal tissue and other major organs were grossly examined

During a necropsy, small intestinal tissue samples (< 3 mm thick) were collected from each piglet. The specimens were fixed with 10% formalin for 24 h at room temperature (RT) and embedded in paraffin according to standard laboratory procedures. The formalin-fixed paraffin-embedded tissue samples were cut into 5- to 8-μm thick sections on a microtome (Leica, Wetzlar, Germany), floated in a 40 °C water bath containing distilled water, and transferred to glass slides. The tissue was then deparaffinized in xylene for 5 min and washed with decreasing concentrations of ethanol (100%, 95%, 90%, 80%, and 70%) for 3 min each. Deparaffinized intestinal tissue sections were stained with hematoxylin and eosin (H&E; Sigma, St. Louis, MO) to observe histopathological changes or analyzed using immunohistochemistry (IHC) to detect PEDV antigens with a monoclonal antibody specific for the PEDV N protein (Novogene, China), as described previously.

### Statistical analysis

GraphPad Prism 8 software was used for statistical analysis and figure generation. All the data are expressed as the means ± SDs. Differences between groups were examined for statistical significance *via* mixed-effects analysis or one-way analysis of variance (ANOVA) with Tukey’s multiple comparison post hoc test. The asterisks in the figures indicate significant differences, with *p* < .05 (*p < .05; **p < .01; ***p < .001; ****p < .0001; ns, not significant).

DecisionLinnc1.0 software was employed for ­correlation analysis of survival and antibodies. (https://www.statsape.com/). DecisionLinnc1.0 is a platform that integrates multiple programming language environments and enables data processing, data analysis, and machine learning through a visual interface.

## Results

### The statistics of PEDV outbreaks on farms

**Figure 1.**
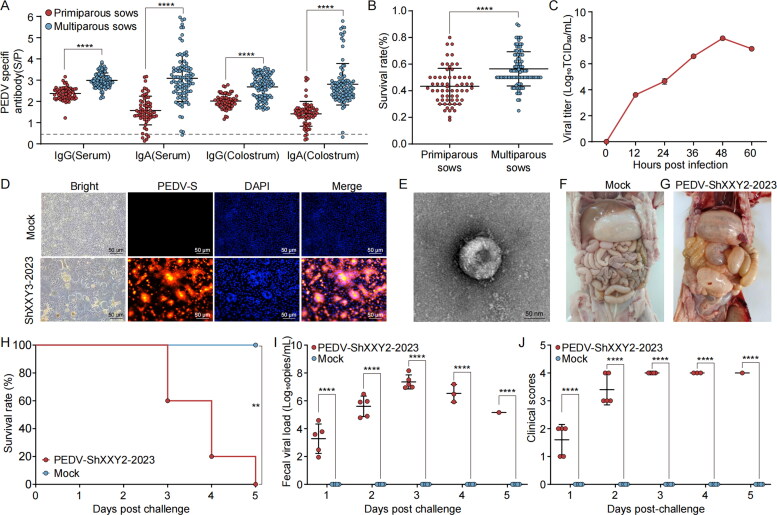
Outbreak farm epidemiology statistics and identification of the isolated PEDV ShXXY2-2023. (A) Serum IgG, IgA antibody and colostrum IgG in sows. (B) Survival of piglets after an outbreak of PEDV on farms. (C) Growth kinetics of the strain PEDV ShXXY2-2023. (D) Vero cells were infected with PEDV isolates for 72 h and produced significant CPE. Indirect immunofluorescence of the infected Vero cells. Vero cells were infected with the obtained PEDV isolates (blue: DAPI, yellow: PEDV) Vero cells were not infected with PEDV used as negative controls. (E) Electron micrograph of PEDV virions in cell culture media of infected Vero cells or on the cell surface of infected Vero cells. Images of PEDV virions from cell culture media of Vero cells infected with the PEDV ShXXY2-2023 strain, as shown by the arrow. Scale bar = 50 nm. Gross anatomy and organ lesions of piglets in the mock and infected groups, normal(left) (F) and diseased(right) (G). (H) Survival rate curve of piglets. (I) PEDV viral load in anal swabs. (J) piglet diarrhea scores.

Most control measures for PEDV currently rely on vaccination. However, the emergence of new PEDV strains has led to recurring diarrhea outbreaks in pig farms (Gao et al. 2021). In January 2023, many piglets suffered from diarrhea and death on a farm in northwestern China. After testing, the diarrhea-related pathogen was determined to be PEDV. Over a period of approximately one month, a total of 62 primiparous sows, 634 piglets, 97 multiparous sows and 1096 piglets were infected with PEDV on the farm. The survival rate of piglets born to primiparous sows was 43%, and the survival rate of piglets born to multiparous sows was 56% (Figure 1(B)). ELISA is commonly used on farms to determine the efficacy of vaccine immunization by testing the S/P value of serum IgG and IgA antibodies in pregnant sows, with a S/P value of >0.5 being considered positive. The results indicated that the antibody levels in both serum and colostrum exceeded the positive threshold. In primiparous sows, the average serum IgG and IgA values were 2.37 and 1.57, respectively, while the average colostrum IgG and IgA values were 2.02 and 1.41, respectively. In multiparous sows, the mean serum IgG and IgA values were 2.98 and 3.09, respectively, and the mean colostrum IgG and IgA values were 2.68 and 2.81, respectively (Figure 1(A)). Despite these positive IgG and IgA antibody results, a significant number of piglets still succumbed to severe diarrhea, vomiting, and weight loss. Clinically healthy piglets free of PEDV infection (Figure S1(A,E,F)) exhibited smooth fur (Figure S1(B,C)) and were housed in a clean environment (Figure S1(D)). In contrast, piglets in affected pens showed signs of infection, with the pigsty notably contaminated by yellow-green, watery feces (Figure S1(G–J)), and dissected intestines displaying considerable flatulence (Figure S1(L)). Additionally, sows also experienced diarrhea (Figure S1(K)).

### Isolation and identification of the PEDV ShXXY2-2023 and the pathogenicity in piglets

PEDV was isolated from the small intestines of pigs with positive RT-qPCR results from a PED outbreak farm. Following three blind passages, Vero cells infected with PEDV exhibited clear cytopathic effects (CPEs), such as cell detachment and aggregation, 48 h post-inoculation, whereas mock-infected cells showed no CPEs. Immunofluorescence with anti-PEDV monoclonal antibodies confirmed the PEDV isolation, revealing specific orange signals in PEDV-infected Vero cells but not in mock-infected cells (Figure 1(D)). After isolation, PEDV was continuously cultured in Vero cells and designated PEDV ShXXY2-2023. Prior to further identification, we performed three rounds of plaque purification in Vero cells, and the titer of the twelfth-generation virus was determined to be 10^7^ TCID_50_/mL.

The infectious titer of the cell-adapted virus ranged from 10^3.61^ to 10^7.96^ TCID_50_/mL (Figure 1(C)). PEDV particles of the PEDV ShXXY2-2023 in Vero cell culture medium and in infected Vero cells were imaged *via* transmission electron microscopy (TEM). TEM revealed that the virions were round and approximately 100 nm in diameter, with surface petal-like fibrils that are characteristic of coronaviruses (Figure 1(E)).

The pathogenicity of cell-cultured PEDV ShXXY2-2023 from passage 12 was demonstrated in the piglets. We randomly divided 10 three-day-old piglets into two groups and orally vaccinated them with PEDV ShXXY2-2023 at a dose of 1 × 10^6^ PFU/mL (1 mL/piglet) or DMEM (DMEM; 1 mL/piglet). The challenged piglets were monitored daily for clinical signs, fecal status, and fecal virus shedding. Daily clinical observations of five challenged piglets revealed mild diarrhea at two days post-infection (dpi), progressing to severe or watery diarrhea between 3 and 5 dpi (Figure 1(J)). The RT-qPCR results of the fecal samples showed that the challenge group had the highest viral load at three days post-challenge, with an average viral shedding of 10 ^7.345^ genomic copies/mL (Figure 1(I)). The death of piglets in the challenged group started on day 3 and all piglets died on day 5, with a death rate of 100% (Figure 1(H)). Pathological autopsy of the overall condition of the intestines showed normal intestines without diarrhea in the PBS group (Figure 1(F)), whereas the intestines of the tapping group were distended and filled with yellow-green feces (Figure 1(G)). The small intestines of the two groups of piglets were collected for pathological section observation. Hematoxylin–eosin staining analysis revealed no obvious lesions in the duodenum or colon of the control group. In the infected group, duodenal vacuole formation and increased lymphocyte levels were observed, jejunal and ileal intestinal villi atrophied and sloughed, and mucosal epithelial degeneration, vacuolization and necrosis occurred (Figure S2). IHC analysis *via* anti-PEDV N protein monoclonal antibodies revealed that the duodenum, jejunum, and ileum of piglets challenged with PEDV ShXXY2-2023 were negative for PEDV, and those of the control group were also negative (Figure S3). These results indicate that the PEDV ShXXY2-2023 is infectious and highly pathogenic to piglets.

### Sequence analysis of the isolated PEDV ShXXY2-2023

**Figure 2.**
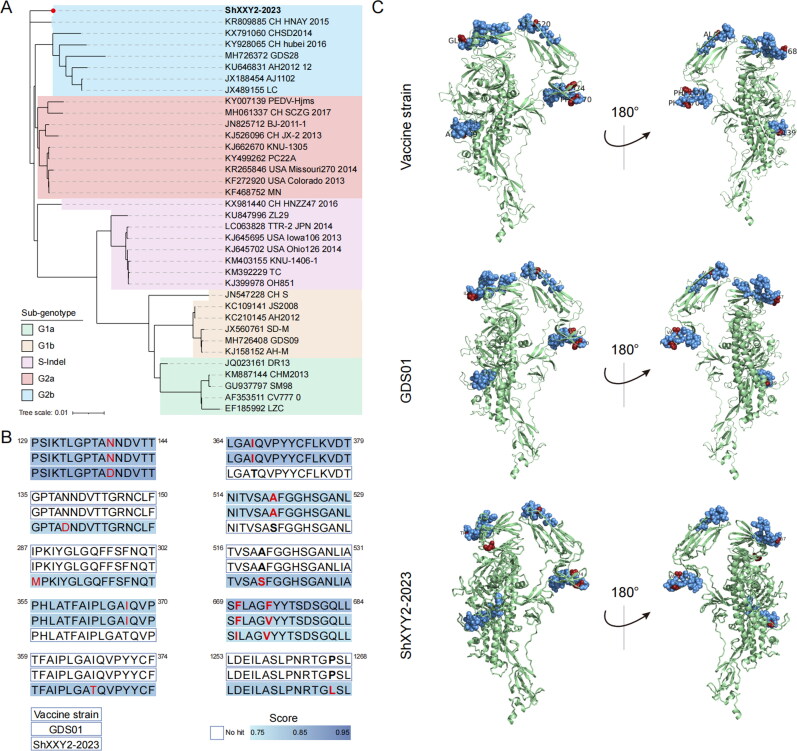
S amino acid sequence analysis and homology modeling of S protein of vaccine strain, PEDV GDS01, PEDV ShXXY2-2023 and prediction of B cell linear epitopes. (A) Phylogenetic analysis of PEDV ShXXY2-2023 based on nucleotide sequences of the Spike genome. Display of B cell linear epitope prediction results on the spatial structure predicted by the SWISS-MODEL program. Red represents the same predicted epitope, blue represents different. (B) Comparison of B-cell linear epitope prediction results for Vaccine strain, PEDV GDS01 and PEDV ShXXY2-2023. C The spatial structures of the S protein were obtained from PEDV vaccine strain, PEDV GDS01, and PEDV ShXXY2-2023. Blue represents the same B cells linear epitopes. The red color represents the sites of different B-cell linear epitopes.

The S protein of PEDV is crucial for binding to specific receptors and eliciting the production of neutralizing antibodies. To date, six neutralizing epitope domains of the S protein have been identified, including S10 (aa 19–220) (Li C et al. 2017), S1A (aa 435–485) (Chang CY et al. 2019), equivalent collagenase domain (COE aa 499–638) (Wang X et al. 2017), SS2 (aa 748–755), and SS6 (aa 764–771) in the S1D region (Sun D et al. 2008), as well as the C-terminal epitope 2C10 (aa 1368–1374) (Lee S et al. 2017; Li C et al. 2017). Viruses with different S protein structures exhibit varying degrees of pathogenicity. The S protein is susceptible to mutation or recombination under natural conditions or selective antibody pressure, which contributes to changes in virulence (Jang et al. 2023). S gene sequence comparison identified the strain as G2b type (Figure 2(A)). Multiple analyses of the deduced S gene amino acids revealed that most variations occurred at the N-terminus of the S protein. Compared with vaccine strains, B-cell linear epitope prediction showed that six amino acid substitutions, including N139D, I287M, I367T, A520S, F670I, F674V and P1265L (Figure 2(B)), affected linear epitopes, such as S10 (aa 19–220), N139D, and COE (aa 499–638) (A520S). With scores >0.8 suggesting potential B-cell linear epitopes. All predicted linear B-cell epitopes were visualized using 3D modeling in PyMOL software. In the structural representation, predicted epitopes are highlighted in blue, while distinct epitopes are marked in red (Figure 2(C)). The analysis revealed that amino acid mutations within these epitopes led to structural alterations in the spike (S) protein. These structural changes suggest that the newly isolated PEDV ShXXY2-2023, exhibits genetic differences from existing vaccine strains, potentially impacting vaccine efficacy.

### A commercial inactivated vaccine protects piglets against the PEDV GDS01 but not the PEDV ShXXY2-2023

Vaccine-induced immunity is the key to preventing PEDV infection (Song D et al. 2015; Sun D et al. 2016; Gerdts and Zakhartchouk 2017). Pregnant sows are typically administered two doses of an inactivated vaccine prior to delivery to ensure the production of high antibody levels, which are transferred to piglets through milk. We conducted a comparative study using a commercial vaccine, followed by challenge with both the PEDV ShXXY2-2023 or PEDV GDS01, to assess the vaccine’s protective efficacy against different PEDV strains. In accordance with standard farm procedures, sows were immunized with two doses of a commercial inactivated vaccine at five and three weeks before delivery (Figure S4(A)). ELISA results showed a significant increase in PEDV-specific IgG and IgA antibody levels in the serum of sows after vaccination (Figure S4(B,C)). The vaccines induced IgG and IgA antibodies in the serum at 14 days post-immunization, with further increases in antibody titers observed at 28 days post-immunization showing a statistically significant difference compared to the PBS group. Newborn piglets from immunized sows were orally inoculated with either PEDV ShXXY2-2023 or PEDV GDS01 at 1 × 10^6^ PFU/mL (1 mL/piglet) at three to five days of age, while piglets from the control group were orally inoculated with 1 mL PBS. The results revealed a low survival rate of 58% in the PEDV ShXXY2-2023 group, an 84% survival rate in the PEDV GDS01 group, and the highest survival rate of 92% in the control group (Figure S4(D)). RT-qPCR analysis of fecal samples showed an average viral shedding of 10^5.66^ genomic copies/mL in the PEDV ShXXY2-2023 group, with the highest viral load occurring at four days post-challenge. In contrast, the highest viral load in the PEDV GDS01 group was 10^2.64^ genomic copies/mL at three days post-challenge. The overall virus shedding was lower in the PEDV GDS01 group compared to the PEDV ShXXY2-2023 group (Figure S4(F)). At three days post-infection, piglets challenged with PEDV ShXXY2-2023 exhibited watery diarrhea, while those challenged with PEDV GDS01 showed only mild diarrhea (Figure S4(E)). Despite the immunization of sows, the different strains led to significantly different survival rates in the piglets. These findings suggest that the commercial vaccine is effective in protecting against the PEDV GDS01 strain but does not provide cross-protection against the PEDV ShXXY2-2023 in piglets.

### Preparation and antibody evaluation of inactivated vaccines

**Figure 3.**
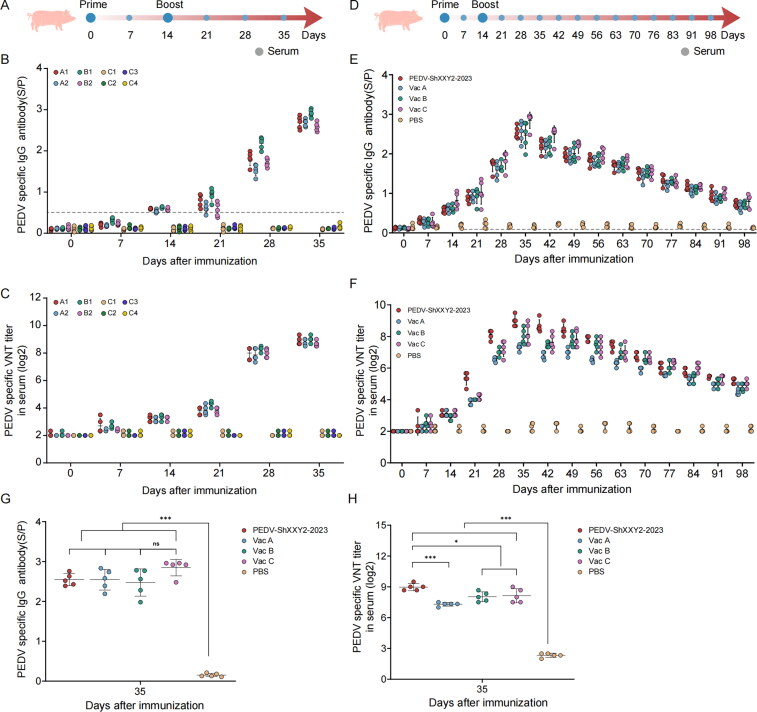
Preparation and antibody assessment of the PEDV ShXXY2-2023 inactivated vaccine. (A) Schematic diagram of immunization program. Serum from different immune groups were collected at designated times to detect IgG (B) and neutralizing antibodies levels (C). (D) Schematic diagram of immunization program. Serum IgA (E) and IgG (F) antibodies were tested at seven-day intervals after immunization and were detected up to 98 days after immunization. Comparison of serum IgG (G) and IgA (H) antibodies 35 days after immunization.

Inactivated vaccines are favored for their safety and ease of production, though their immunogenicity can be reduced during the inactivation process, often necessitating multiple doses or booster shots to achieve effective immunity (Park 2024). We first assessed the immunogenicity of the PEDV ShXXY2-2023 formulated as an inactivated vaccine using different adjuvants and inactivation methods, as outlined in Table 1, with an antigen concentration of 10^7^ TCID_50_/mL. A 2 mL dose was administered *via* intramuscular injection into the neck (Figure 3(A)). Antibody titers were measured prior to immunization and at 7,14,21,28 and 35 days post-immunization. The results indicated that groups A1, A2, B1, and B2 elicited high levels of IgG and neutralizing antibodies (Figure 3(B,C)). Three weeks after the second immunization, the S/P values for IgG antibodies in the A1, A2, B1, and B2 groups were 2.688, 2.682, 2.916, and 2.582, respectively; neutralizing antibodies against PEDV ShXXY2-2023 titers were 1:467, 1:436, 1:512, 1:407 respectively. Therefore, we chose the inactivation method and adjuvant of group B1 for subsequent experiments.

To compare the immunogenicity of the PEDV ShXXY2-2023 inactivated vaccine with that of commercially available inactivated G2 vaccines, 25 PEDV-free, four-week-old piglets were randomly assigned to five groups (5 piglets per group) and housed separately. The immunization schedule and challenge timeline are depicted in Figure 3(D). Piglets in the experimental groups received 2 mL of the PEDV ShXXY2-2023 inactivated vaccine or one of the commercial inactivated G2 vaccines (Vac A, Vac B, Vac C) *via* intramuscular injection into the neck, with a booster dose administered 14 days after the initial immunization. The control group received 2 mL of PBS. Serum samples were collected at seven-day intervals until 98 days post-immunization to measure IgG and neutralizing antibody levels against PEDV ShXXY2-2023.The results showed that all four vaccine groups induced high levels of IgG antibodies, which peaked on day 35 post-immunization, with IgG S/P values of 2.546, 2.546, 2.474, and 2.848 for the PEDV ShXXY2-2023, Vac A, Vac B, and Vac C groups, respectively (Figure 3(E)). No significant differences were observed among the vaccine groups (Figure 3(G)). Neutralizing antibody titers also peaked on day 35, with the mean titers for the PEDV ShXXY2-2023, Vac A, Vac B, and Vac C groups reaching 1:495, 1:156, 1:261, and 1:276, respectively (Figure 3(F)). Notably, a significant difference in neutralizing antibody levels was observed in response to the PEDV ShXXY2-2023 challenge (Figure 3(H)). The lower neutralizing antibody levels produced by the three commercial vaccines against the PEDV ShXXY2-2023 strain may explain their reduced cross-protection against emerging variants.

### The PEDV ShXXY2-2023 inactivated vaccine effectively protects newborn piglets from PEDV infection, while commercial vaccines demonstrate lower efficacy

PEDV causes mainly severe diarrhea in newborn piglets, resulting in high mortality and causing enormous economic losses to the pig industry (Song D and Park 2012; Jung and Saif 2015; Goede and Morrison 2016). One key reason for the susceptibility of newborn piglets to viral infections is their underdeveloped immune system. As a result, passive immunity is the most effective form of protection against viral infections. After sows are immunized with effective PEDV vaccines, their piglets receive protective antibodies through colostrum and milk, providing passive immune protection (Bohl et al. 1972). The experiments demonstrated that while different vaccines produce similar levels of IgG antibodies, significant differences in neutralizing antibody titers exist. Therefore, we assessed the protective effects of different vaccines by challenging piglets born to sows immunized with various vaccines to determine if differences in neutralizing antibodies contributed to the variation in protection.

**Figure 4.**
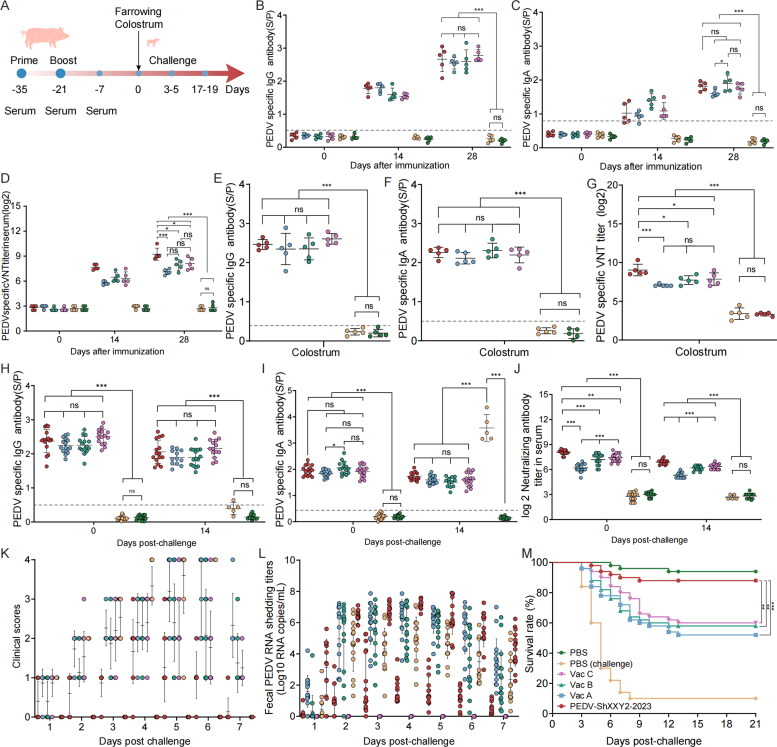
Antibody detection in sows vaccinated with inactivated vaccines and assessment of challenge protection in piglets (challenge with PEDV ShXXY2-2023). (A) Schematic diagram of immunization program. (B) Specific IgG titers in sow serum. (C) Specific IgA titers in sow serum. (D) Sow serum neutralizing antibody titers against PEDV ShXXY2-2023. (E) specific IgG titers in sow colostrum. (F) Specific IgA titers in sow colostrum. (G) Sow colostrum neutralizing antibody titers against PEDV ShXXY2-2023. (H) Detection of serum IgG antibodies in piglet before and after challenge. (I) Detection of serum IgA antibodies in piglet before and after challenge. (J) piglet serum neutralizing antibody titers against PEDV ShXXY2-2023 before and after challenge. (K) Clinical scores of each group. The clinical scores of individual pigs from each group was measured as described in the Materials and Methods section. (L) Fecal PEDV shedding profile of each group. PEDV RNA titers (log10 genomic copies/ml) in rectal swaps at the indicated sampling time points were determined using RT-qPCR. (M) Piglet survival rate after challenge.

The sow immunization procedure followed the protocol outlined in Figure 4(A). We measured IgG, IgA, and neutralizing antibodies in the serum of vaccinated pregnant sows, as well as sIgA, SIgG, and neutralizing antibodies in their milk. Additionally, we assessed serum IgG, IgA, and neutralizing antibodies in the piglets, both before and after the viral challenge, as well as fecal virus shedding, clinical symptoms, and piglet survival rates. Similar to the piglet vaccination experiments, the titers of IgG (Figure 4(B)) and IgA antibodies (Figure 4(C)) in sow serum and colostrum (Figure 4(E,F)) increased significantly after the booster immunization, with no significant differences between groups. However, compared to the PEDV ShXXY2-2023 group, the neutralizing antibody titers against PEDV ShXXY2-2023 in the serum and colostrum of sows vaccinated with commercial inactivated Vac A, Vac B, and Vac C were significantly lower (Figure 4(D,G)). Specifically, at two weeks post-booster, the neutralizing antibody titers in serum and colostrum were 1: 610 and1: 523 for the PEDV ShXXY2-2023 group; 1: 138 and1: 135 for Vac A; 1: 247 and 1: 221 for Vac B; and 1: 273 and 1: 232 for Vac C, respectively.

Piglets were challenged with the PEDV ShXXY2-2023 at three days old and monitored daily for clinical signs, fecal status, and virus shedding. Serum samples were collected before and 14 days post-challenge for IgG, IgA, and neutralizing antibody analysis. On the first day post-challenge, vomiting occurred in the PBS (challenge), Vac A, Vac B, and Vac C groups. Diarrhea began on the second day, with only a few piglets in the PEDV ShXXY2-2023 group showing mild symptoms. In contrast, piglets in the Vac A, Vac B, and Vac C groups exhibited widespread diarrhea and vomiting, while the PBS (challenge) group displayed severe diarrhea, vomiting, and dehydration (Figure 4(K)). RT-qPCR analysis of fecal samples revealed that the average viral shedding on day 4 was 10^1.61^, 10^6.37^, 10^5.5^, 10^4.98^, and 10^7.2^ genomic copies/mL for the PEDV ShXXY2-2023, Vac A, Vac B, Vac C, and PBS (challenge) groups, respectively. The PEDV ShXXY2-2023 group had significantly lower viral loads compared to the three commercial vaccine groups and the PBS (challenge) group (Figure 4(I)). Notably, the PBS group did not exhibit diarrhea or other symptoms.

Piglets in the PBS (challenge) group and the Vac A, Vac B, and Vac C groups began to experience mortality starting on the third day of the challenge. On the 21st day post-challenge, the survival rate of piglets in the PBS control group was 94% (47/50), while the survival rate for the PEDV ShXXY2-2023 group was 88% (44/50). In contrast, the survival rates for piglets in the Vac A, Vac B, and Vac C groups were 52% (26/50), 58% (29/50), and 60% (30/50), respectively. The survival rate in the PBS (challenge) group was only 10% (5/50) (Figure 4(M)). The survival rate in the PEDV ShXXY2-2023 group was similar to that of the PBS group. In comparison, the commercial vaccine groups had survival rates ranging from 52% to 60%. These findings indicate that despite the commercial vaccines generating high levels of IgG and IgA antibodies, their protective efficacy for passively immune piglets against the PEDV ShXXY2-2023 is insufficient.

We measured IgG, IgA, and neutralizing antibodies against PEDV ShXXY2-2023 in piglet serum before and 14 days after the challenge. Serum antibody levels in all piglet groups correlated with those in sows, although they were slightly lower in piglets. Antibody levels decreased gradually over time (Figure 4(H,1,J)). At 14 days post-challenge, serum IgG levels in the PBS (challenge) group were essentially undetectable. In contrast, very high levels of IgA antibodies were observed in the serum of PBS (challenge) piglets that survived 14 days post-challenge. These IgA levels were significantly higher than those in the four vaccine groups. The mean serum IgA antibody S/P ratio in all vaccine groups was 1.61, whereas it reached 3.58 in the PBS (challenge) group. In the sera of the five surviving piglets, neutralizing antibody titers were approximately 1:4 and IgG antibodies were undetectable. These findings suggest that PEDV infection can induce a strong IgA-mediated immune response within a short period of time in piglets.

Despite different vaccines inducing high levels of IgG and IgA antibodies in sows, and consequently high levels of these antibodies in passively immunized piglets, challenge with the PEDV ShXXY2-2023 resulted in varying degrees of diarrhea and high mortality among piglets. While IgG and IgA antibody levels showed no significant differences across vaccine groups, neutralizing antibody levels varied significantly. Piglets with higher neutralizing antibody titers exhibited milder clinical symptoms and higher survival rates. Thus, neutralizing antibody levels serve as a crucial indicator of a vaccine’s protective efficacy.

### Vaccination of pregnant sows with the PEDV ShXXY2-2023 inactivated vaccine protects piglets against the PEDV GDS01 strain

**Figure 5.**
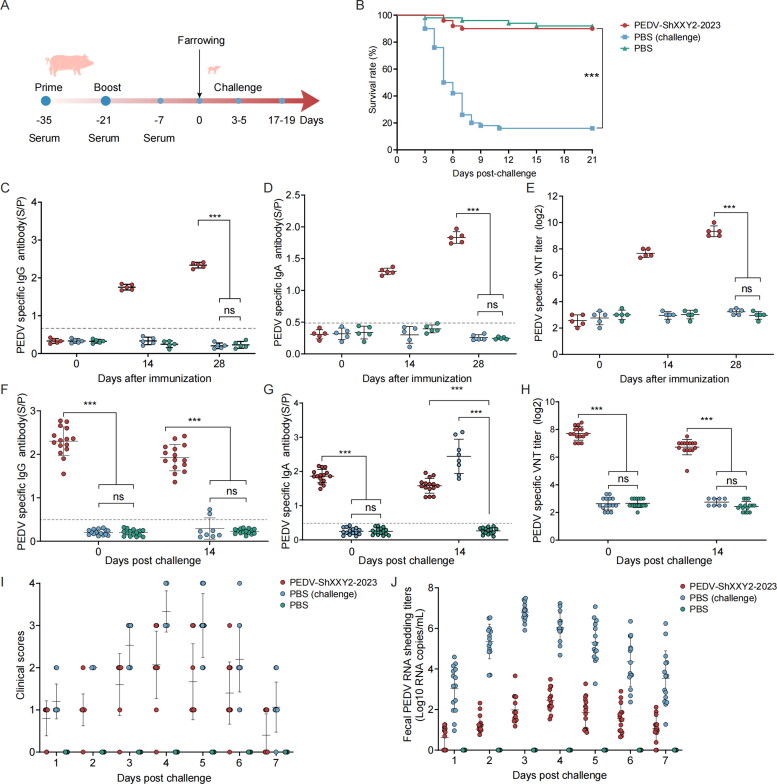
The protective efficacy of the inactivated PEDV ShXXY20202 strain vaccine in sows against PEDV GDS01 strain infection in piglets. (A) Schematic diagram of immunization program. (B) Piglet survival rate after challenge. (C) Specific IgG titers in sow serum. (D) Specific IgA titers in sow serum. (E) Sow serum neutralizing antibody titers against PEDV GDS01. (F) Detection of serum IgG antibodies in piglet before and after challenge. (G) Detection of serum IgA antibodies in piglet before and after challenge. (H) Piglet serum neutralizing antibody titers against PEDV GDS01 before and after challenge. (I) Clinical scores of each group. The clinical scores of individual pigs from each group were measured as described in the Materials and Methods section. (J) Fecal PEDV shedding profile of each group. PEDV RNA titers (log10 genomic copies/ml) in rectal swaps at the indicated sampling time points were determined using RT-qPCR.

The results confirmed that the PEDV ShXXY2-2023 inactivated vaccine provided effective protection against the PEDV ShXXY2-2023. Consequently, we evaluated the efficacy of this vaccine by immunizing sows and subsequently challenging their piglets with the PEDV GDS01. The immunization and challenge procedures followed the protocol shown in Figure 5(A). The findings indicated that piglets immunized with the PEDV ShXXY2-2023 vaccine had a survival rate of 90% (45/50), while piglets in the PBS group had a survival rate of 92% (46/50). In contrast, the survival rate for piglets in the PBS (challenge) group was only 16% (8/50) (Figure 5(B)). Following vaccination, sows developed high levels of IgG, IgA, and neutralizing antibodies against the PEDV GDS01 (Figure 5(C,D,E)). The piglets exhibited passive immunity through colostrum and milk consumption, with high levels of IgG, IgA, and neutralizing antibodies detected in their serum (Figure 5(F,G,H)). After the PEDV GDS01 challenge, piglets from the vaccine group remained healthy with no signs of diarrhea, vomiting, or other symptoms. Clinical scores and viral load measurements in swabs were lower compared to those in the control virus challenge group (Figure 5(I,J)). These results demonstrate that the PEDV ShXXY2-2023 vaccine effectively protects piglets from PEDV GDS01 challenges.

### Neutralizing antibodies, rather than IgG or IgA, are the most crucial factor determining the survival of piglets following PEDV challenge

**Figure 6.**
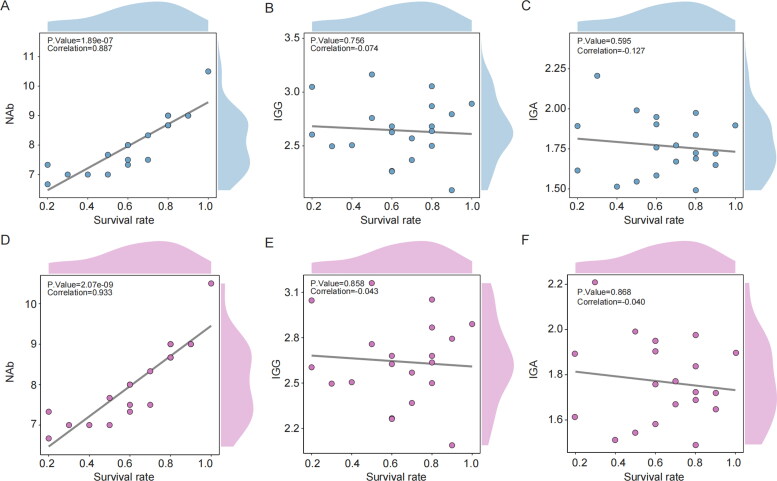
The relationship between IgA, IgG, neutralizing antibodies and survival rate. (A, D) Correlation between neutralizing antibodies and survival. (B, E) Correlation between IgG and survival. (C, F) Correlation between IgA and survival.

Immunizing pregnant sows with vaccines provides significant protection to their piglets through passive immunity acquired *via* colostrum consumption (Langel et al. 2016). To evaluate this, we collected serum samples from four groups of sows that had been administered inactivated vaccines and measured IgG, IgA, and neutralizing antibodies two weeks after the second immunization. We then compared the survival rates of their piglets following viral challenge and performed Pearson correlation analyses to assess the relationship between neutralizing antibody levels and survival rates. The analysis demonstrated a strong positive correlation between neutralizing antibody levels and piglet survival, r (17) = 0.887 (Figure 6(A)). In contrast, IgG r (17) = 0.0741 (Figure 6(B)) and IgA r (17) = 0.127 (Figure 6(C)) levels showed no significant correlation with survival. This correlation is also supported by the spearman correlation coefficient (Figure 6(D,E,F)). These results indicate that higher neutralizing antibody levels in the serum of late-pregnancy sows are strongly associated with increased piglet survival after viral challenge. Descriptive statistical analysis was conducted to evaluate the relationship between maternal serum neutralizing antibody levels two weeks post-vaccination and the survival rate of neonatal piglets following PEDV challenge. Prior to formal analysis, descriptive statistics were performed to characterize the dataset. This procedure generates standardized summary tables (three-line tables), which facilitate rapid visualization of data distribution, trends, and other key features. In this module, a binary or multiclass categorical variable must first be selected as the independent (grouping) variable, followed by the selection of continuous or categorical variables to be described. This module serves as a vital tool for the efficient interpretation and summarization of large datasets. In this analysis, the grouping variable was set as the survival rate of piglets after PEDV challenge, and the variable subjected to statistical description was the maternal neutralizing antibody titer. Continuous variables were presented in the format of Mean (95% CI). The resulting summary table reported the mean, minimum, maximum, 95% confidence interval, and standard deviation for each continuous variable, along with the frequency and percentage for categorical variables. The p-values were derived from comparisons of group means using either t-tests (for two groups) or ANOVA (for more than two groups). The results demonstrated that when maternal serum log2 neutralizing antibody titers ranged from1:377 to 1:774 or higher, over 80% protection was conferred to piglets against PEDV infection (Table 2). These findings suggest that neutralizing antibody levels can be used as a predictive marker for determining the protective efficacy of vaccines administered to pregnant sows, and may serve as an early indicator of sufficient maternal immunity.

**Table 2.** Predicting the relationship between serum neutralizing antibodies and piglet survival in pregnant sows one week before farrowing.

Variable names	Overall	Missing	0	1	*p*	SMD
*N*	20		12	8		
Antibody_value	8.092 (7.639,8.545)	0.0	7.444 ([7.131,7.758])	9.064 ([8.56,9.568])	<.001	2.940

### Neutralizing antibodies are a key factor in protection against viral infections

**Figure 7.**
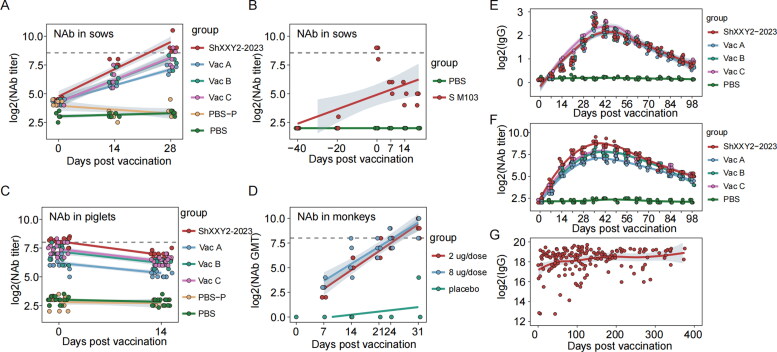
Comparison of coronavirus neutralizing antibodies and post-infection antibody protection correlation. (A) Serum neutralizing antibodies against PEDV ShXXY2-2023 in pregnant sows immunized with different PEDV vaccines. (B) Serum neutralizing antibody levels in pregnant sows immunized with PDCoV vaccine. (C) Serum neutralizing antibodies in piglets born to pregnant sows immunized with different PEDV vaccines against PEDV ShXXY2-2023. (D) Serum neutralizing antibody levels in monkeys immunized with SARS-CoV-2 vaccine. (E) Serum IgG antibody levels in four-week-old piglets immunized with different PEDV vaccines against PEDV ShXXY2-2023. (F) Serum neutralizing antibody levels in four-week-old piglets immunized with different PEDV vaccines against PEDV ShXXY2-2023. (G) Serum IgG antibody levels in humans immunized with SARS-CoV-2 vaccine.

Protective correlations have been identified for various virus-infected hosts. For some viruses and vaccines, the kinetics of the antibody response are well understood, allowing for accurate predictions of the duration of protection. These correlations are typically based on specific antibody levels acquired through vaccination or natural infection, which can significantly reduce the risk of (re)infection (Wajnberg et al. 2020). For instance, a hemagglutination inhibition titer of 1:40 for influenza virus reduces the risk of infection by 50% (Krammer et al. 2020). Similar titers have been established for measles virus (ID50 titer of 1:200), hepatitis A virus, and hepatitis B virus (Plotkin 2010). These titers have greatly facilitated vaccine development (Van Herck and Van Damme 2001). For coronaviruses, the Food and Drug Administration recommends minimum thresholds for antibody activity in recovering plasma (e.g. NT50 > 160) (Salazar et al. 2020). In trials against Porcine deltacoronavirus (PDCoV), neutralizing antibodies greater than 1:407 in sow serum at farrowing provided effective protection for piglets (Figure 7(B)) (Li J et al. 2023). This result generally supports our prediction that protection against PEDV is mediated by neutralizing antibodies (Figure 7(A)). Two doses of the BBIBP-CorV vaccine, 2 μg and 8 μg, have been reported to be highly effective against SARS-CoV-2 in rhesus monkeys, with neutralizing antibody levels of 1:215 and 1:256, respectively, detected 10 days post-vaccination. No antibody-dependent enhancement (ADE) or worsening of immunopathology was observed, indicating that neutralizing antibody titers effectively prevent SARS-CoV-2 infection(Figure 7(D)) (Wang H et al. 2020). In this study, piglets acquired neutralizing antibodies from colostrum provided by sows. Sows in the PEDV ShXXY2-2023 group produced the highest levels of neutralizing antibodies, with an average titer of 1:272 in their newborn piglets (Figure 7(C)). In contrast, piglets from the Vac A, Vac B, and Vac C groups had average neutralizing antibody titers of 1:74, 1:147, and 1:169, respectively (Figure 7(C)). These three groups produced lower levels of protective neutralizing antibodies, which may contribute to severe diarrhea in piglets infected with the PEDV ShXXY2-2023.

We compared the serum IgG and neutralizing antibody levels after immunization with the PEDV vaccine to those after immunization with the SARS-CoV-2 vaccine. We found that both PEDV and SARS-CoV-2 immunizations resulted in sustained antibody levels (IgG) (Figure 7(E,G)). SARS-CoV-2 produced longer-lasting antibody levels, possibly because the subjects were previously infected with SARS-CoV-2 before vaccination, leading to prolonged antibody levels. We also observed that the duration of changes in IgG and neutralizing antibody levels was consistent (Figure 7(F)). These results suggest that there is a certain consistency in the patterns of antibody dynamics following coronavirus vaccination.

### Piglet challenge protection test for pregnant sows with high and low levels of neutralizing antibodies

**Figure 8.**
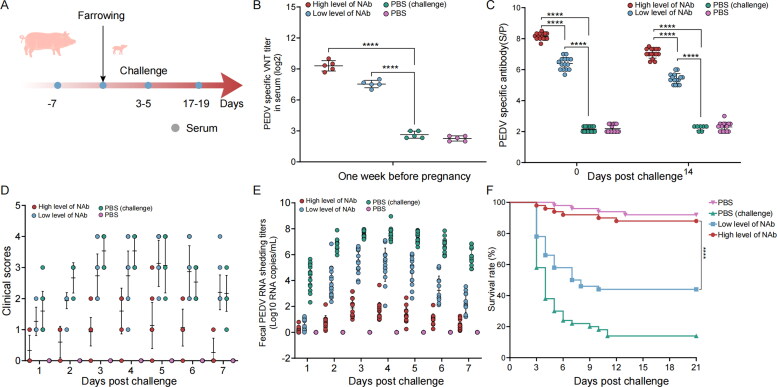
Evaluation of the protective effect of sows with high and low levels of neutralizing antibodies against piglet challenge. (A) Schematic diagram of immunization program. (B) Sow serum neutralizing antibody titers against PEDV ShXXY2-2023. (C) Piglet serum neutralizing antibody titers against PEDV ShXXY2-2023 before and after challenge piglet survival rate after challenge. (D) Clinica scores of each group. (E) Fecal PEDV shedding profile of each group. PEDV RNA titers (log10 genomic copies/ml) in rectal swaps at the indicated sampling time points were determined using RT-qPCR. (F) Piglet survival rate.

We predicted that serum neutralizing antibody titers in sows, measured during the week before farrowing, between 1:377 and1:774 would provide 80% protection against PEDV infection in piglets. Consequently, we selected pregnant sows with high and low antibody levels for validation (Figure 8(A)), and detected the serum antibody titers in the week before farrowing in the high level neutralizing antibody group as 1:1024, 1:724, 1:400, 1:512, 1:637; and in the low level neutralizing antibody group as 1:256, 1:200, 1:181, 1:128, 1:181 (Figure 8(B)).

The antibody levels in newborn piglets were comparable to those in their sows, though slightly lower by approximately one titer (Figure 8(C)). After the challenge, piglets in the high-antibody group exhibited mild diarrhea, with no signs of vomiting or severe diarrhea. In contrast, the low-antibody group and the PBS (control) group showed severe diarrhea and death, while piglets in the PBS group remained healthy and free of diarrhea (Figure 8(D)). RT-qPCR analysis of fecal samples revealed average viral shedding on the fourth day post-challenge, with genomic copies/mL at 10^1.606^, 10^5.71^and 10^7.52^ in the high-antibody, low-antibody, and PBS groups, respectively (Figure 8(E)). At 21 days post-challenge, the survival rate of piglets in the high-antibody group was 88% (44/50), compared to 44% (22/50) in the low-antibody group, and 14% (7/50) in the PBS (challenge) group. In the PBS control group, the survival rate was 92% (46/50). These findings confirm that the high-antibody group provided effective protection against the PEDV ShXXY2-2023 (Figure 8(F)), consistent with our prediction that neutralizing antibody levels are a key indicator of PEDV immunoprotection.

## Discussion

The development of vaccines with high immunogenicity and safety is crucial for controlling PEDV and preventing further disease and mortality. Since PEDV primarily infects neonatal piglets, passive immunization through sow vaccination plays a vital role in protecting them from infection. Newborn piglets acquire antibodies, mainly IgG and sIgA, by consuming colostrum and milk, which allows these antibodies to enter the piglets’ bloodstream and mucosal surfaces to provide systemic and local protection. This mechanism is referred to as the gut-mammary-sIgAaxis. Therefore, the primary goal of sow vaccination is to induce high levels of both systemic and mucosal antibodies that can be effectively transferred to piglets to prevent systemic and intestinal PEDV infections (Bohl et al. 1972).

Although vaccines are currently the most effective strategy for controlling coronaviruses, including PEDV, their efficacy is often compromised by the emergence of mutant strains. Genetic mutations, including insertions and deletions, enhance viral adaptation and may alter the structure and function of viral proteins involved in pathogenicity, infectivity, transmission, and antigenicity. These factors result in antigenic drift, which reduces the cross-protective efficacy of existing vaccines against heterologous strains, particularly the newly emerging G2b variants that exhibit immune evasion due to mutations in neutralizing epitopes. The spike (S) glycoprotein is the key viral protein that determines host receptor binding and tissue tropism, making it a major target for diagnosis, antiviral therapy, and vaccine design. Numerous mutations, particularly deletions in the S1 N-terminal domain (S1-NTD), have been observed in the evolution of coronaviruses. Most NTD mutations have been found to alter antigenicity or eliminate epitopes, aiding immune evasion (Harvey et al. 2021; Khateeb et al. 2021). Such as K417N/T found in B.1.351 and Gamma (P.1) was also found to evade antibody binding, though less potent than E484 substitutions (Barton et al., 2021; Wang P, Casner, et al., 2021; Wang P, Nair, et al., 2021). These findings emphasize the challenge of maintaining vaccine efficacy against rapidly evolving variants. Therefore, future vaccination strategies must focus on enhancing neutralizing antibody responses against circulating variants to ensure broad protection (Emary et al. 2021; Kunal et al. 2021; Wang H et al. 2023).

Our study addresses several of these challenges by developing an inactivated vaccine based on the PEDV variant strain PEDV ShXXY2-2023. This strain contains amino acid substitutions in key neutralizing epitopes within the NTD, receptor-binding domain (RBD), and CO-26K equivalent epitope (COE) regions of the S1 subunit. These regions are known to harbor critical neutralizing epitopes. Structural modeling and comparative alignment suggest that these mutations may alter the surface topology and electrostatic properties of the epitopes, thereby reducing the binding affinity of neutralizing antibodies induced by existing vaccines. Recent studies further support this notion, demonstrating that conformational changes within specific regions of the S1 subunit – such as the COE region (aa 498–638) and the domain 0 (D0) of the NTD – are closely associated with diminished cross-neutralization capacity and enhanced viral adaptability (Zhang Y et al. 2022; Luo et al. 2024). These findings provide molecular evidence that mutations in the PEDV-ShXXY2-2023 strain may contribute to immune evasion by altering the accessibility or presentation of neutralizing epitopes, ultimately leading to reduced vaccine efficacy.

The PEDV ShXXY2-2023 inactivated vaccine developed in this study demonstrated superior efficacy compared to current commercial inactivated vaccines in reducing both mortality and diarrhea severity. In contrast, commercial inactivated vaccines (Vac A, Vac B, and Vac C), although capable of inducing IgG and IgA responses, failed to elicit sufficient neutralizing antibody titers against the PEDV ShXXY2-2023. Consequently, these commercial inactivated vaccines did not provide adequate protection for piglets, as evidenced by lower survival rates and increased fecal viral shedding observed during the challenge studies. Notably, maternal serum-neutralizing antibody titers exceeding1:377 one week before farrowing were strongly correlated with improved piglet survival rates post-challenge. This underscores the critical role of maintaining high maternal neutralizing antibody levels in providing effective protection for neonatal piglets.

Additionally, previous studies have reported a correlation between IgG, sIgA, and neutralizing antibody responses (Song X et al. 2023). However, in our study, such correlation was observed only in the neutralizing antibodies induced by the PEDV ShXXY2-2023 inactivated vaccine, which showed a significant association with both IgG and sIgA levels. In contrast, no significant correlation was found between neutralizing antibodies and IgG or sIgA in sows immunized with the commercial inactivated vaccines A, B, and C. This discrepancy may be attributed to the differing neutralization efficacy of these commercial vaccines against the PEDV ShXXY2-2023. It may result from variations in the S proteins used in the PEDV IgG/sIgA ELISA kits and those present in the strains employed for neutralization assays, thereby affecting the observed relationship between neutralizing antibodies and IgG or sIgA levels.

In addition, the results of this study showed that the inactivated PEDV ShXXY2-2023 vaccine induced sustained IgG and neutralizing antibody responses, with protective antibody titers maintained for up to 98 days post-immunization, suggesting that the inactivated vaccine is capable of eliciting long-term protective humoral immunity. Interestingly, five piglets from the PBS (challenge) group survived for 14 days post-challenge. Their sera tested negative for IgG and neutralizing antibodies, but showed strong IgA positivity. Since antibody levels were not assessed in the other piglets of the PBS (challenge) group on day 14, it remains unclear whether the survival of these five piglets was due to a rapid IgA response shortly after infection that provided transient protection, or if it was the result of individual variation. Further animal studies are required to clarify this observation.

Our findings highlight the necessity for next-generation PEDV vaccines that prioritize the induction of potent neutralizing antibodies targeting conserved epitopes, while also enhancing mucosal immunity. Future vaccine development strategies should focus on selecting vaccine strains representative of currently circulating field variants and incorporating antigens targeting conserved neutralizing epitopes to broaden cross-protection. Additionally, integrating mucosal adjuvants may further enhance sIgA responses and improve mucosal immune defense in neonatal piglets. Exploring novel vaccine platforms, including subunit vaccines, viral vectored vaccines, and mRNA-based vaccines, may also offer improved immunogenicity and safety profiles compared to traditional inactivated or live attenuated vaccines (Guo et al., 2024; Zhao et al. 2024).

In conclusion, our study demonstrates the superior efficacy of the PEDV ShXXY2-2023 inactivated vaccine in inducing high titers of neutralizing antibodies and providing broad protection against homologous and heterologous PEDV strains. These findings emphasize the critical role of neutralizing antibodies in conferring protection against PEDV infection in neonatal piglets. The strategies and insights described in this study provide a valuable foundation for the rational design and development of effective PEDV vaccines and may also inform vaccine development efforts for other coronaviruses with significant implications for animal and public health.

## Supplementary Material

Supplemental Material

## Data Availability

The datasets used and/or analyzed during the current study available from the corresponding author on reasonable request.
