# Supplementary material for: Neutralizing antibody levels as a key factor in determining the immunogenic efficacy of the novel PEDV alpha coronavirus vaccine
Source: Vet Q. 2025 May 28;45(1):1–20. doi: 10.1080/01652176.2025.2509506 (PMC12120861; doi:10.1080/01652176.2025.2509506)
Supplement: Supplemental Material [file TVEQ_A_2509506_SM3090.zip › suppl_data/Supplementary Material.docx]

**Fig. S1 Clinical symptoms in piglets following an outbreak of PEDV on an infected farm.**

**a -f** Clinical signs of healthy piglets, **a** Anal without diarrhea symptoms. **b** No vomiting in piglets. **c** No diarrheal feces on the piglet's body. **d** No diarrheal feces in the pig house. **e** No diarrhea in sows. **f** Dissected piglets with normal intestinal tract. **g-l** Clinical symptoms in piglets infected with PEDV, **g** Yellowish-green Feces in the anus. **h** Vomiting in piglets. **i** Thinning piglets. **j** Pig houses filled with diarrheal feces. **k** Diarrhea in sows. **l** Dissecting the intestinal tract of infected piglets.

**Fig. S2 Immunohistochemistry (IHC) analysis (20X magnification) of the experimentally challenged animals. Hematoxylin and eosin-stained tissue sections of the jejunum from ShXXY2-2023-infected and mock control piglets**

**Fig. S3 Immunohistochemistry (IHC) analysis (20X magnification) of the experimentally challenged animals. Detection of PEDV antigens via IHC analysis of jejunal tissue sections from ShXXY2-2023-infected and mock control piglets**

**Fig. S4 Comparison of GDS01 and ShXXY2-2023 administration in sows challenged piglets after vaccination.**

In the experimental design, the sows were randomly divided into 3 groups (n=5). Pregnant sows were immunized with the commercial inactivated vaccine 5 weeks and 2 weeks before delivery, and the control sows were immunized with PBS. Serum was collected during immunization and two weeks after immunization to quantify PEDV-specific IgA and IgG by ELISA. On the 3rd to 5th days after the birth of each litter, 10 piglets were randomly selected and challenged with ShXXY2-2023 or GDS01 (6 log10 PFU/mL; 1 mL/piglet). The control group was orally administered 2 mL of DMEM. After the challenge, the survival rate and clinical symptoms of the piglets were monitored, and the viral copy number of swabs was determined (**a**). Monitoring of serum PEDV-specific IgG (**b**) and IgA (**c**) levels by ELISA in pregnant sows before and after vaccination. Survival rate (**d**), clinical score (**e**)and viral copy number in swabs of 3- to 5-day-old newborn piglets challenged with ShXXY2-2023 or GDS01 after sows were vaccinated with 2 doses of vaccine (**f**). Error bars indicate the standard deviation. * p < 0.05; ** p < 0.01; *** p < 0.001; ns, not significant.
