# Supplementary figures and images for: Neutralizing antibody levels as a key factor in determining the immunogenic efficacy of the novel PEDV alpha coronavirus vaccine
Source: Vet Q. 2025 May 28;45(1):1–20. doi: 10.1080/01652176.2025.2509506 (PMC12120861; doi:10.1080/01652176.2025.2509506)

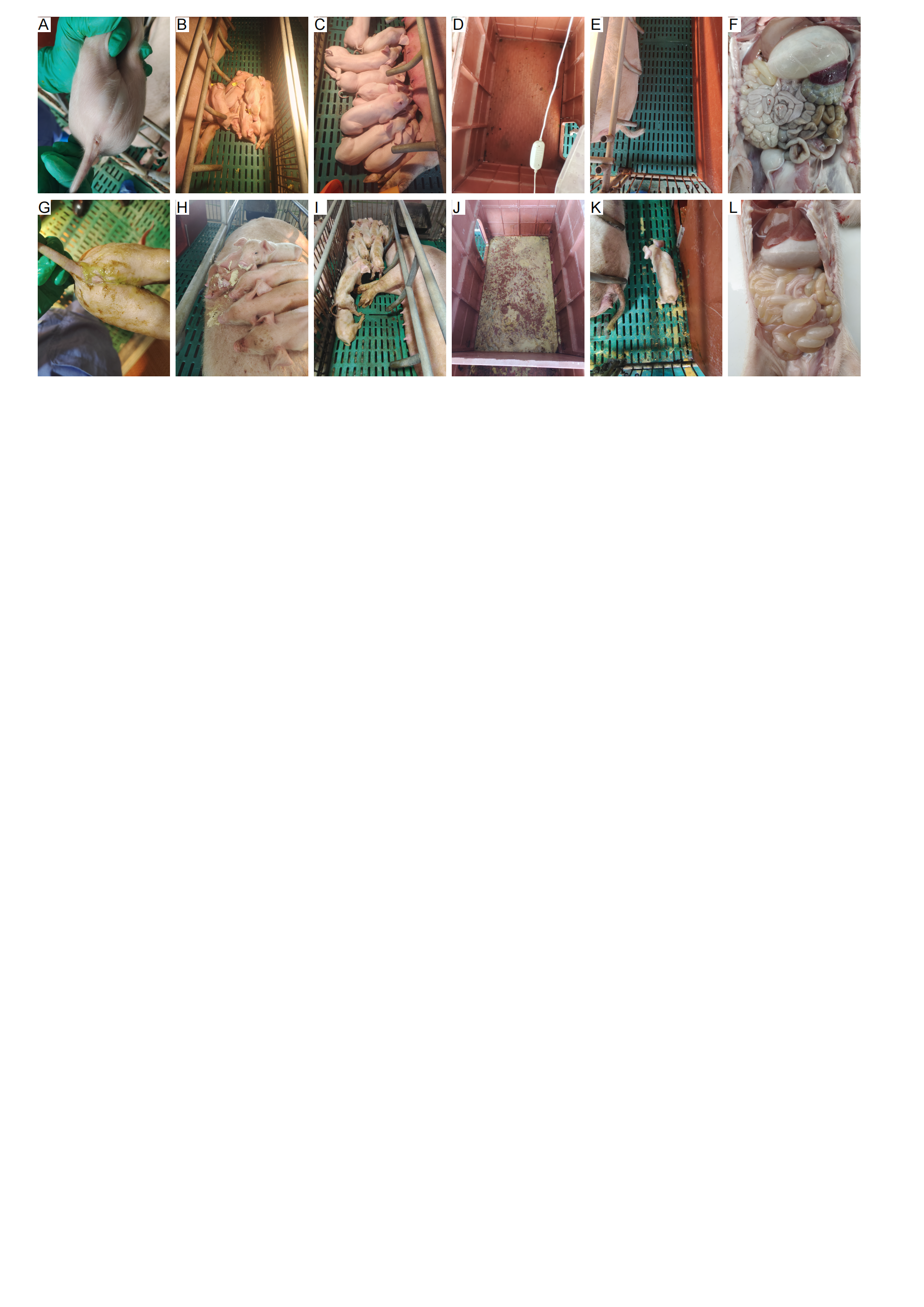

Supplement: Supplemental Material [file TVEQ_A_2509506_SM3090.zip › suppl_data/FigureS1_00.tif]

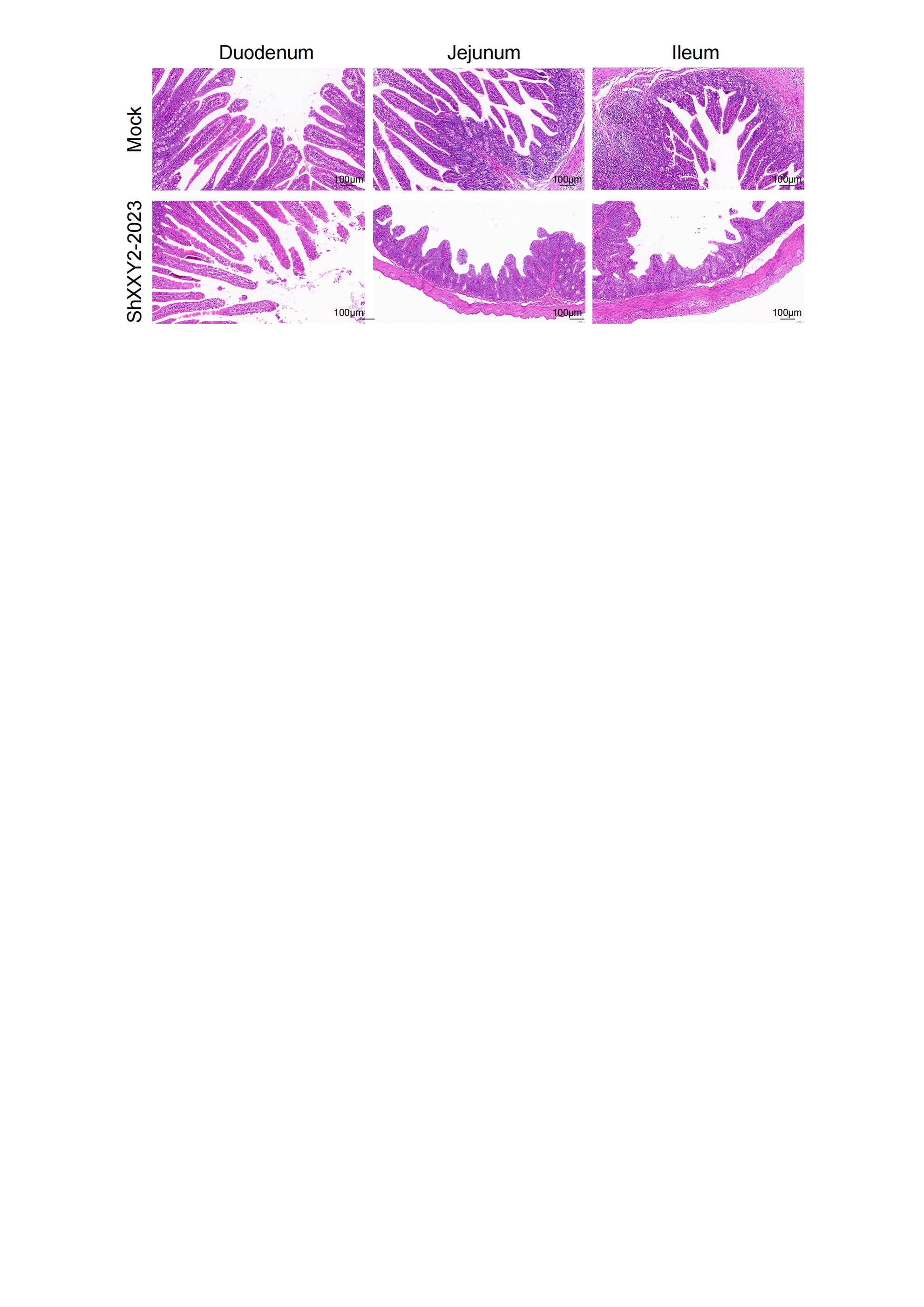

Supplement: Supplemental Material [file TVEQ_A_2509506_SM3090.zip › suppl_data/FigureS2_00.tif]

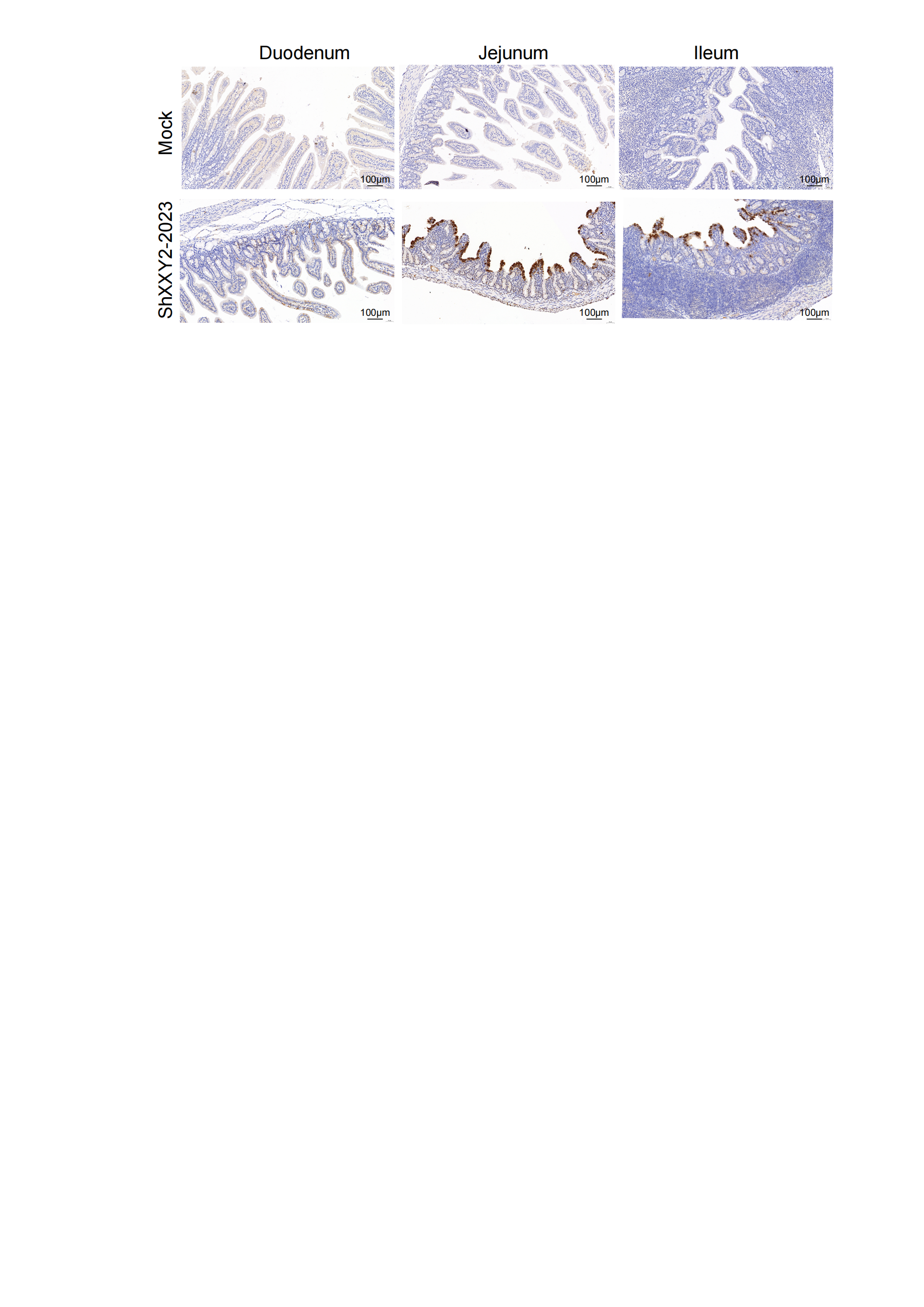

Supplement: Supplemental Material [file TVEQ_A_2509506_SM3090.zip › suppl_data/FigureS3_00.tif]

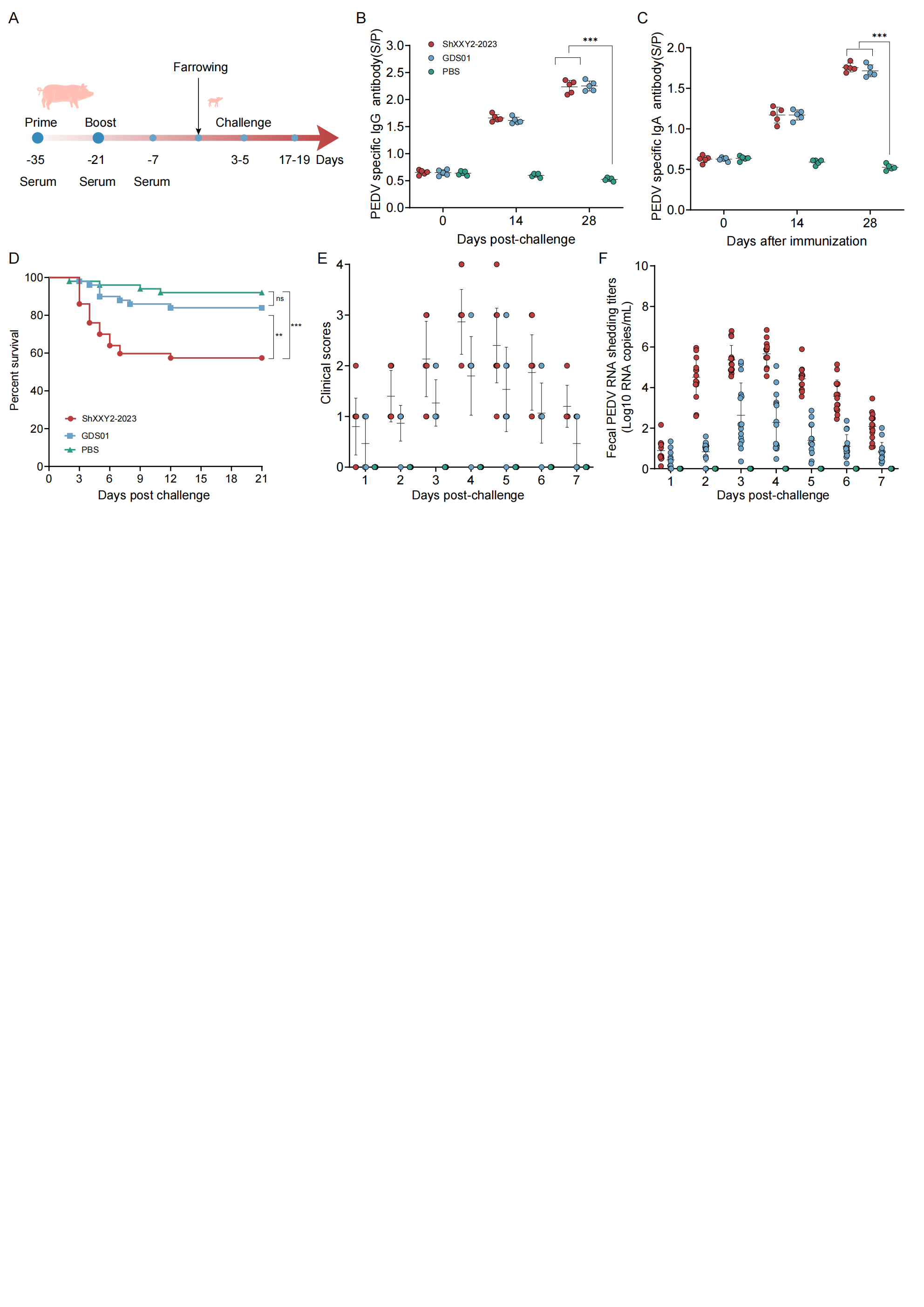

Supplement: Supplemental Material [file TVEQ_A_2509506_SM3090.zip › suppl_data/FigureS4_00.tif]
